# A New Stress Test for Knee Joint Cartilage

**DOI:** 10.1038/s41598-018-38104-2

**Published:** 2019-02-19

**Authors:** Chinmay S. Paranjape, Hattie C. Cutcliffe, Steven C. Grambow, Gangadhar M. Utturkar, Amber T. Collins, William E. Garrett, Charles E. Spritzer, Louis E. DeFrate

**Affiliations:** 1 Department of Orthopaedic Surgery, Duke University, Durham, NC USA; 2 Department of Biomedical Engineering, Duke University, Durham, NC USA; 3 Department of Biostatistics and Bioinformatics, Duke University, Durham, NC USA; 4 Department of Radiology, Duke University, Durham, NC USA; 5 Department of Mechanical Engineering and Materials Science, Duke University, Durham, NC USA

## Abstract

Cartilage metabolism—both the synthesis and breakdown of cartilage constituents and architecture—is influenced by its mechanical loading. Therefore, physical activity is often recommended to maintain cartilage health and to treat or slow the progression of osteoarthritis, a debilitating joint disease causing cartilage degeneration. However, the appropriate exercise frequency, intensity, and duration cannot be prescribed because direct *in vivo* evaluation of cartilage following exercise has not yet been performed. To address this gap in knowledge, we developed a cartilage stress test to measure the *in vivo* strain response of healthy human subjects’ tibial cartilage to walking exercise. We varied both walk duration and speed in a dose-dependent manner to quantify how these variables affect cartilage strain. We found a nonlinear relationship between walk duration and *in vivo* compressive strain, with compressive strain initially increasing with increasing duration, then leveling off with longer durations. This work provides innovative measurements of cartilage creep behavior (which has been well-documented *in vitro* but not *in vivo*) during walking. This study showed that compressive strain increased with increasing walking speed for the speeds tested in this study (0.9–2.0 m/s). Furthermore, our data provide novel measurements of the *in vivo* strain response of tibial cartilage to various doses of walking as a mechanical stimulus, with maximal strains of 5.0% observed after 60 minutes of walking. These data describe physiological benchmarks for healthy articular cartilage behavior during walking and provide a much-needed baseline for studies investigating the effect of exercise on cartilage health.

## Introduction

Cartilage metabolism is related to the mechanical loading of the tissue because chondrocytes, the cells responsible for maintaining cartilage architecture, are mechanically sensitive^1–7^. Cyclic or dynamic loading of cartilage *in vitro* stimulates chondrocyte anabolism, with increased biosynthesis seen in explants^3^, chondrocytes seeded within agarose^4,8,9^, and in tissue-engineered cartilage constructs^10^. Conversely, static loading *in vitro* results in decreased biosynthesis in similar models^4,11^. Further, animal studies of joint immobilization show decreases in cartilage stiffness^12^, a mechanical property of the tissue, with remobilization helping to restore stiffness^13^. These observations suggest that mechanical loading is necessary for the maintenance of cartilage structure and health^14^.

Therefore, exercise is often recommended as a potential treatment for osteoarthritis (OA), a disabling disease of synovial joints that results in cartilage degeneration^2^. In controlled trials of exercise intervention for OA, physical activity has resulted in decreased pain scores^15,16^, decreased disability scores^15,16^, and increased glycosaminoglycan content as measured by delayed gadolinium-enhanced magnetic resonance imaging^17^. However, it is still unclear how exercise modifies cartilage biomechanics and ultimately joint health. As such, mapping the appropriate exercise duration and intensity to specific therapeutic targets cannot currently be performed because knowledge of how different types and intensities of exercise influence the mechanical environment of the joint is limited. Detailed dose-response studies quantifying changes in cartilage mechanical response with changes in the duration and intensity of exercise are needed to fill this gap.

Cartilage mechanical response can be quantified by measuring either stress or strain within the tissue^18–20^, which are related to one another by the tissue’s mechanical properties (e.g. stiffness). Under a constant stress, cartilage displays creep behavior^21–23^, in which the strain increases over time. Leveraging this time-dependent (viscoelastic) response of cartilage to mechanical load^24^, our laboratory has recently developed a new technique for investigating cartilage strain *in vivo*^18^ using three dimensional (3D) modeling and magnetic resonance imaging (MRI). This approach provides a means to evaluate cartilage function by applying an exercise stimulus—an *in vivo* stress—and measuring the resulting strain response, analogous to a cardiac stress test. Therefore, our cartilage stress test may be used to quantify tissue health: as cartilage strain response depends on its mechanical properties and composition, both of which are altered in OA^25^, the local strain response may be different in OA patients versus healthy individuals. Furthermore, by measuring strain via MRI, our cartilage stress test can be applied noninvasively to a variety of populations. As walking is the most common form of exercise in the US^26^, we explored the dose-dependent effects of walk duration and walk speed on cartilage thickness and strain, a necessary step to inform exercise-based OA therapies.

Specifically, we quantified the effects of varying durations and speeds on the *in vivo* response of cartilage following walking exercise to develop a cartilage stress test in a dose-dependent manner. This investigation provides important insight into normal *in vivo* cartilage biomechanics under varying intensities of loading, which has important implications for OA progression, prevention, and treatment. Additionally, this work provides innovative measurements of normative cartilage creep during walking, revealing this fundamental mechanical behavior in healthy subjects. Overall, this study addresses core questions about cartilage mechanics and informs exercise-based treatment approaches to slowing the progression of OA.

## Results

**Figure 1:**
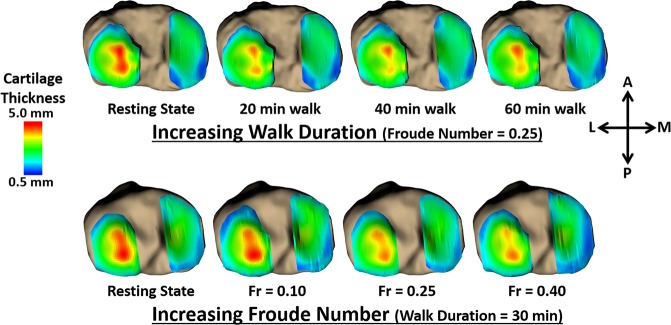
Representative tibial cartilage thickness maps (superior-inferior perspective of axial view of left knee) for the first protocol (top row; increasing walk duration) and for the second protocol (bottom row; increasing normalized walking speed); increased duration and increased normalized speed each resulted in decreased cartilage thickness compared to the baseline resting state, with thickness changes leveling off with longer walk durations. Averaged across all volunteers, pre-exercise (baseline) cartilage thickness was 2.5 ± 0.3 mm (mean ± 95% confidence interval), 2.1 ± 1.8 mm, and 3.0 ± 2.6 mm across the entire tibial plateau, the medial plateau, and the lateral plateau, respectively.

In order to measure the mechanical response of cartilage to walking, we compared cartilage thickness distributions across the tibial plateau obtained using MR imaging before and after bouts of walking exercise at different durations and speeds. To apply consistent loading between subjects, walk speed was normalized using the Froude Number (Fr)^27^. Increased walk duration at a fixed normalized speed (Fr = 0.25) resulted in decreased cartilage thickness (Fig. 1) and thus increased compressive strain. Increased normalized speed for a fixed walk duration (30 minutes) also resulted in decreased cartilage thickness (Fig. 1) and increased compressive strain.

**Figure 2:**
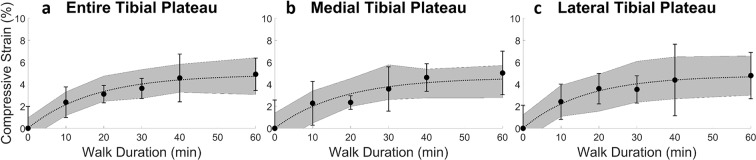
Overall (**a**) and compartmental (**b**,**c**) compressive strain (mean, 95% confidence interval) in tibial cartilage as a function of walk duration, for a fixed normalized walking speed (Fr = 0.25); sample size (n) = 10 for all time points except 60 min where n = 8; compressive strain significantly increased (Spearman correlation, p < 0.05) with increasing walk duration in a nonlinear fashion (lines represent the nonlinear mixed effects model fits of the Kelvin-Voigt two-parameter creep model to the duration data while shading represents the 95% prediction interval for the median of this fit).

As walk duration increased (for a fixed walking speed, Fr = 0.25), mean tibial cartilage compressive strain increased monotonically (never decreased), eventually plateauing at longer walk durations (Fig. 2). This asymptotic pattern of increased compressive strain with increased walk duration was consistent across the entire tibial plateau (Fig. 2a), and when the medial and lateral portions were analyzed separately (Fig. 2b and c). Spearman correlation indicated that the increase in compressive strain with increased walk duration was statistically significant (p < 0.05) for the entire tibial plateau (p = 0.001) and for each compartment (medial: p = 0.002, lateral: p = 0.04). Additionally, compressive strain as a function of walk duration was well-fit by the Kelvin-Voigt exponential model for creep (Fig. 2, see Supplementary File for model diagnostics). After 10 minutes of walking, compressive strain (mean ± 95% confidence interval) was 2 ± 1%, 2 ± 2%, and 2 ± 2% for the overall tibia, medial plateau, and lateral plateau, respectively; while for 20 minutes of walking it was 3 ± 1%, 2 ± 1%, and 4 ± 1%, respectively. Compressive strains in the tibial cartilage leveled off to 5 ± 2%, 5 ± 2%, and 5 ± 2% (overall tibia, medial plateau, and lateral plateau, respectively) at 60 minutes of walking.

**Figure 3:**
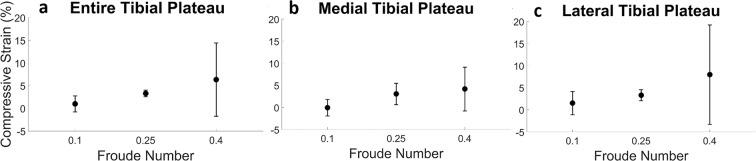
Overall (**a**) and compartmental (**b**,**c**) compressive strain (mean, 95% confidence interval) in tibial cartilage as a function of normalized walking speed (Froude Number, Fr), for a fixed walk duration (30 minutes); sample size (n) = 7 for Fr = 0.1, n = 10 for Fr = 0.25, n = 8 for Fr = 0.40; compressive strain significantly increased (linear mixed model, p < 0.05) with increased normalized walking speed for the overall tibia and medial tibial plateau, but not for the lateral tibial plateau.

As normalized walking speed increased (for a fixed duration of 30 minutes), mean tibial cartilage compressive strain increased from a slow pace (Fr = 0.10), to a moderate pace (Fr = 0.25), and to a fast pace (Fr = 0.40; close to the walk-run transition of Fr = 0.5^27^) (Fig. 3). This pattern was consistent across the entire tibial plateau (Fig. 3a), and when the medial and lateral compartments were analyzed separately (Fig. 3b and c). Linear mixed modeling indicated that Fr was a significant predictor for the overall tibial strain (p = 0.004) and medial plateau strain (p = 0.03), but not the lateral plateau strain (p = 0.15). At Fr = 0.10, compressive strain (mean ± 95% confidence interval) was 1 ± 2%, 0 ± 2%, and 2 ± 3% for the overall tibia, medial plateau, and lateral plateau, respectively. At Fr = 0.25, compressive strain was 3 ± 1%, 3 ± 2%, and 3 ± 1%, respectively (overall, medial, lateral); while for Fr = 0.40, compressive strain was 6 ± 8%, 4 ± 5%, and 8 ± 11%, respectively.

## Discussion

As mechanical loading influences chondrocyte metabolism^1–3^, certain levels of exercise or *in vivo* loading may promote cartilage health^14^. Therefore, investigating various exercise intensities on the *in vivo* response of healthy cartilage is a crucial first step toward OA therapies and prevention programs. Towards this end, the current study provides a direct measure of cartilage compressive strain after walking, an activity of daily living and also a common exercise, in a duration- and speed-dependent manner. We used a combination of MR imaging and site-specific 3D modeling of subjects’ cartilage before and after activity to investigate bouts of walking at different durations and speeds, in order to quantify the resultant cartilage compressive strain^18–20^. Our walk duration results demonstrate that for a fixed, comfortable walking speed, as walk duration increased, cartilage compressive strain across the tibial plateau increased monotonically (Spearman correlation, p < 0.05). Moreover, compressive strain approached an asymptote between forty and sixty minutes of walking. Finally, our results also demonstrate that for a fixed walk duration, as normalized walking speed increased, overall tibial cartilage and medial tibial plateau compressive strain also increased (linear mixed model, p < 0.05).

Our observations of an asymptotic relationship between increasing walk duration and cartilage strain demonstrate *in vivo* creep. Cartilage creep behavior has been well-documented *in vitro*^21–23^, but not *in vivo*. Creep occurs due to the motion of fluid within the tissue: initially, there is a high degree of fluid loss at the tissue surface, where there is a high amount of strain. Over time, the fluid loss near the surface of the cartilage redistributes throughout the rest of the tissue until an equilibrium strain and loss of fluid is reached. This redistribution levels off in an exponential manner across time^21^. Importantly, the *in vivo* cartilage strains observed in the present study demonstrated similar behavior, illustrated by the agreement between the Kelvin-Voigt exponential model^28^ and our strain versus walk duration data (Fig. 2, see Supplementary File for model diagnostics). Since *in vitro* work has demonstrated a link between mechanical loading and chondrocyte metabolism^3,4,8–11^, our observations of cartilage creep occurring *in vivo* are vital for leveraging *in vitro* findings towards preventing, treating, and ameliorating OA degeneration.

Prior work including *in vitro* studies, animal studies, and exercise intervention studies has pointed towards exercise as a potential therapeutic or preventative measure for OA^13,14,17^. Therefore, the design of this study—a dose-response investigation analyzing both exercise time (walk duration) and exercise intensity (walking speed) on *in vivo* cartilage strain in a site-specific manner—represents a vital piece of information for establishing therapeutic targets and clinical interventions for OA, as it quantifies the response of healthy cartilage to physiological loading (various bouts of walking). This normative data is crucial for informing future studies, as it provides physiological boundary conditions (strain values) to apply during investigations of mechanical loading and cartilage health. Furthermore, despite walking being the most popular form of exercise in the US^26^, there is a lack of data in the literature characterizing cartilage response to walking. A previous study from our lab investigated the cumulative *in vivo* cartilage deformation after only a single bout of walking at a fixed duration^18^, while the present study quantified cartilage deformation after multiple bouts of walking at different durations. In addition, the current study also investigated the effect of walking speed. Our speed results support the notion that faster cycles within a set length of time load the cartilage more. We observed that as walking speed increased, so too did the compressive strain in the tibial cartilage (Fig. 3). As increased walking velocity is accompanied by increased ground reaction forces^29^, increased speed likely also elevates joint reaction forces and thus the forces acting on the tibial cartilage. Therefore, faster walking may involve larger joint forces than slower walking, potentially leading to the larger cartilage deformations seen at higher Fr than seen following walking at lower Fr. Lastly, we observed that the compressive strains resulting from walking were not evenly distributed across the cartilage surface (Fig. 1), indicating that site-specific measurements are needed to accurately capture cartilage response across the tissue. As OA may incur focal tissue changes or defects, such as increased thinning in cartilage sub-regions compared to the overall compartment^30^, the ability to capture focal cartilage changes in a site-specific manner may shed further light into OA etiology^31,32^.

Overall, the work presented here offers several implications for OA treatment and prevention. Current therapeutic guidelines for walking as an exercise intervention for OA target symptoms such as pain and physical function^33^. The data in this study is a first step toward developing therapeutic measures aimed at improving cartilage health, as it characterizes compressive strain in healthy cartilage to various degrees of walking exercise (normative tibial cartilage response data). However, future studies are needed to determine the response of degraded or OA cartilage to these intensities of walking, in order to inform therapeutic targets and exercise recommendations. Likewise, the *in vivo* stress test methodology presented here can be used in future work to formulate recommendations for safe levels of several other types of exercise, such as running or cycling, as well as to investigate the response of other cartilage surfaces in the knee, such as the femoral cartilage and the articular surfaces of the patellofemoral joint. The benefits of this noninvasive stress test are that it is both patient-specific and site-specific: our methodology can be used to determine cartilage response on an individual patient basis, and it can detect particular regions of cartilage thinning or local changes in strain. These two features taken together can inform specific therapeutic regimens to target certain degenerative phenotypes. Furthermore, this approach may also be used as an *in vivo* creep test to non-invasively quantify mechanical properties of cartilage. As such, it is a novel way to study the degenerative OA process, as individuals can be followed over time to investigate changes in *in vivo* cartilage mechanical properties with normal aging. OA etiology can further be explored using our stress test by testing healthy controls, populations at high risk of OA (such as with obesity^34^ or prior joint injury^35^), and those already suffering from OA to characterize differences between *in vivo* mechanical properties of healthy, at risk, and OA cartilage. Lastly, combining this methodology with other techniques, such as quantitative MR imaging (e.g. T1rho or T2 mapping^34,36–39^) or blood-based markers, may increase the sensitivity and specificity of OA diagnosis.

In conclusion, this study used a novel cartilage stress test based on MR imaging and 3D modeling to quantify tibial cartilage compressive strain after walking in a dose-dependent manner for both walk duration and speed. Further, this study provided innovative observations of *in vivo* creep (monotonically-increasing compressive strain) in response to increasing walk duration. These *in vivo* observations are consistent with data describing *in vitro* cartilage behavior under compression^21–23,40^, and are also consistent with studies investigating *in vivo* cartilage biomechanics^18,19,41,42^. While treatments for OA are limited, exercise is able to ameliorate pain associated with OA^15,16,33^ and represents a promising intervention capable of influencing cartilage health. However, the appropriate type, intensity, and duration of exercise cannot yet be prescribed as direct *in vivo* evaluation of exercise on cartilage health has not been performed. Towards this end, the data of this study represent physiological benchmarks for healthy cartilage response to walking, which may aid in the design of treatment and prevention strategies for those suffering from OA. Further, the technique presented here provides a framework for future studies investigating the effects of various types of exercise on cartilage health in a variety of populations, such as those at high risk for OA. Both the novel data and innovative methodology of the current study are useful for future work to determine exercise regimens that may be used therapeutically to treat or prevent OA.

## Materials and Methods

### Data Collection

Duke University Health System Institutional Review Board (IRB) approval was obtained prior to starting this study. All study procedures were carried out in compliance with the IRB’s regulations. Ten healthy subjects, including five males and five females (mean age: 25 years, range: 22–27 years; mean BMI: 22.1 kg/m^2^, range: 20.0–24.7 kg/m^2^) with no history of injury or surgery to any of the joints in their lower extremities, provided informed written consent prior to their participation. The same subjects participated in two different walk protocols, each consisting of several separate sessions, where each session took place on a separate day: 1) walking at a fixed, comfortable speed with bouts of different durations (ranging from 10 to 60 minutes) and 2) walking for a fixed duration of 30 minutes at different speeds (including a low, moderate, and high walk speed). Subjects began in the first walk protocol of the study and were given the option to also participate in the second protocol at a later date. The exercise duration or speed was randomized in each protocol.

For each session of both protocols, subjects arrived at 7am, in order to minimize the known diurnal pattern of cartilage strain in the knee and to minimize loading of the joint prior to the study^19^. They were instructed not to perform any strenuous exercises the night before or morning of the study. After arrival, subjects rested supine for 45 minutes prior to a baseline MRI scan^20^. They were transported to the MR scanner (located adjacent to the room where they rested) using a wheelchair to avoid any weight-bearing following the resting period. Imaging was performed using a 3 Tesla scanner (Trio Tim, Siemens Medical Solutions USA, Malvern, PA) with an 8-channel receive-only knee coil. During imaging, subjects lay supine with their knee in a relaxed, extended position. Sagittal plane images (field of view: 16 × 16 cm; resolution: 512 × 512 pixels; slice thickness: 1 mm) were generated using a double-echo steady-state sequence (DESS; flip angle: 25°; repetition time: 17 ms; echo time: 6 ms)^43^. Total pre-activity scan time per session was approximately 9 minutes. Subjects underwent the 45 minutes of rest and pre-activity baseline scan in each study session.

Subjects were then transported via wheelchair to an adjacent treadmill. Under the first protocol, in each session subjects walked for one of five durations (10, 20, 30, 40, or 60 minutes; one duration per session). Subjects also wore a Fitbit (Fitbit Inc., San Francisco, CA) during the walk to quantify step count. Immediately following the walk, subjects underwent a post-activity scan (same as the pre-activity scan; see details above). Time from walk completion to post-activity MRI initiation was 3:24 ± 0:29 minutes (mean ± standard deviation), and ranged from 2:27 to 4:47 minutes. This amount of time is small compared to the reported 90 minutes for patellar cartilage volume^41^ to recover to baseline after activity. In all sessions, subjects walked at a fixed, comfortable walk speed which was constant across sessions. To account for differences in height and leg length, which affect stride length and loading frequency and therefore loading between subjects, speed was normalized to the subject’s leg length using the Froude Number (Fr)^27^, in order to ensure a similar loading level was applied across volunteers. The Froude Number is a dimensionless quantity which takes into account the subject’s limb length (*L*, in m), defined as the vertical distance from the ground to the greater trochanter; the gravitational constant (*g* = 9.81, in m/s^2^); and the subject’s walk velocity (*v*, in m/s)^27^. Limb length was determined by measuring the distance from the greater trochanter (via manual palpation) down to the surface of the floor. For the first protocol, all subjects walked at a normalized speed of Fr = 0.25. For each walk duration, the normalized speed remained constant, meaning that increased walk durations resulted in an increased number of steps.

If subjects chose to participate in the second protocol, the same procedure described above was repeated, except that each session consisted of a fixed 30 minute walk duration at one of three different speeds. Three normalized walking speeds were chosen *a priori* to span possible walking speeds of a human adult^27,44^: Fr = 0.10 (corresponding to 0.9 ± 0.04 m/s for the volunteers in this study, mean ± standard deviation), representing an adult walking with impaired mobility; Fr = 0.25 (corresponding to 1.5 ± 0.04 m/s for the volunteers in this study), representing an adult walking at a comfortable pace; and Fr = 0.40 (corresponding to 1.9 ± 0.09 m/s for the volunteers in this study), representing a walk close to the run-walk transition. Walk velocity (treadmill speed) was then calculated from these Fr, via Equation 1^27^:

1 $$v=\sqrt{(Fr)(L)(g)}$$

For each normalized speed, the walk duration remained constant, meaning that increased normalized speeds resulted in not only increased numbers of steps, but step frequencies as well.

Because our prior study found significant cartilage strain after a single bout of walking using a sample size of eight^18^, we aimed to recruit between eight and ten subjects for the current study. Ten subjects participated in the first protocol, with eight subjects completing all five sessions (two subjects completed all but the 60 minute duration walk). Of the ten, eight subjects chose to participate in the second protocol, with seven of the eight subjects completing all three sessions (one subject did not participate in the slow walk speed, Fr = 0.10).

### Data Analysis

**Figure 4:**
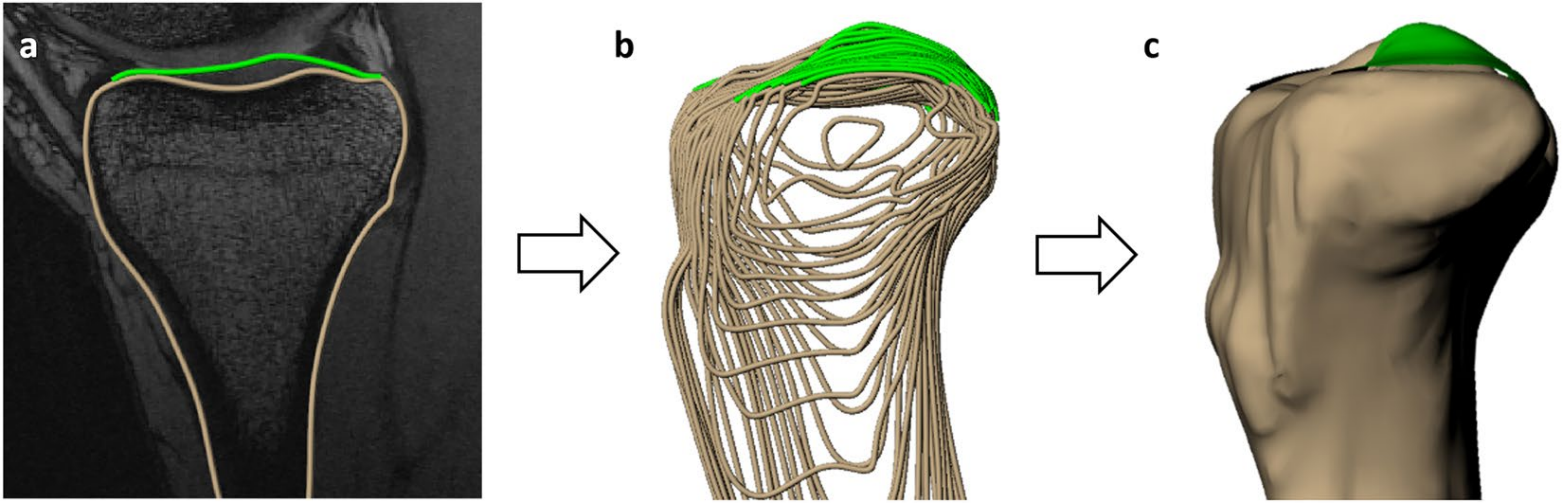
3D joint model creation from MRI (sagittal view, left knee), showing **a**) segmentation of articular cartilage and bone in a single MRI slice, **b**) stacked wireframe model of segmented surfaces, and c) 3D surface mesh model of the tibia and tibial cartilage.

Cartilage thickness was measured via creation of three dimensional (3D) knee joint models from the MR images (Fig. 4). This technique has been previously validated to measure changes in cartilage thickness^45^ and has been shown to have a coefficient of variation of 1%^19^. This methodology has been used to measure diurnal changes in cartilage thickness^19^ as well as changes in cartilage thickness after a single bout of walking^18^. To do so, the boundaries of the tibial cortex and tibial articular cartilage surface were manually traced by a single investigator in each slice of the MR images (Fig. 4a) using solid modeling software (Rhinoceros 4.0, Robert McNeel and Associates, Seattle, WA). The segmentations from each slice were then stacked to create a wireframe model of the joint (Fig. 4b), which was used to generate a 3D surface mesh model (Fig. 4c) of both the bone and cartilage (Geomagic Studio 11, 3D Systems, Rock Hill, SC).

**Figure 5:**
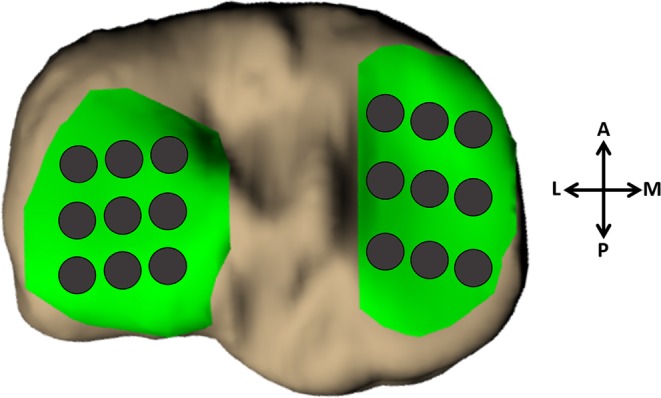
18-point grid point sampling scheme, showing nine evenly-spaced sampling regions on both the medial and lateral tibial plateaus (superior-inferior view, left knee).

The bony surfaces of the pre- and post-activity tibia models were aligned using an iterative closest-point technique (Geomagic Studio 11, 3D Systems, Rock Hill, SC). Then, a grid system of 18 sampling points was applied to measure cartilage thickness in a site-specific manner at the same locations in the pre- and post-activity models. The grid system consisted of nine evenly-spaced sampling points on both the medial and lateral tibial plateaus (Fig. 5). To measure cartilage thickness at each sampling point, custom software (Wolfram Mathematica 9, Wolfram Research, Champaign, IL) was used^18^. First, cartilage thickness, defined as the distance between each point on the cartilage surface mesh and the nearest point on the bony surface mesh, was calculated. Then, the thickness values within a 2.5 mm radius of each sampling point were averaged to define the cartilage thickness at that sampling point. Subsequently, compressive strain was defined at each sampling point as the change in thickness from pre- to post-activity, normalized to the pre-activity thickness (positive values indicate compression, corresponding to a decrease in thickness after activity). As data was collected across multiple sessions, the strain resulting from activity during a particular session was calculated with respect to that session’s pre-activity scan. Overall compressive strain was reported as the average strain across all 18 points on the tibial cartilage, with compartmental strains (medial and lateral) similarly defined as the average across the nine medial and nine lateral sampling points, respectively.

Statistical analyses were performed using SAS (SAS 9.4, SAS Institute Inc., Cary, NC) and R^46^ with a significance level of p < 0.05. Data were checked for normality using the Shapiro-Wilk test. Therefore summary statistics (mean, 95% confidence interval) were calculated for overall and compartmental strain values at each duration and at each normalized speed. Two-tailed Spearman correlation was used to determine if a monotonic relationship existed between compressive strain and increasing walk duration. Linear mixed effects (LME) modeling was used to determine whether normalized walk speed was a significant predictor of compressive strain.

Experiments using confined compression^21,23,47–56^ or indentation testing^22,57–62^ have shown cartilage to behave in a time-dependent viscoelastic manner, where cartilage creeps (shows asymptotic increases in strain over time) in response to a constant load. During this creep response, increases in strain level off exponentially with time^21^. Therefore, the empirical Kelvin-Voigt viscoelastic two-parameter exponential model^28^ (Equation 2) was fit to the compressive strain versus walk duration data (Fig. 2). This model was chosen as it is the simplest phenomenological model that captures creep behavior; however, other models (such as the standard linear solid or biphasic models) could alternately be used^28^. Nonlinear mixed effects (NLME) modeling was performed using the saemix package in R^63^.

2 $${\rm{Strain}}=A[1-\exp (\,-\,B(walk\,duration))]$$

Resting baseline thicknesses were obtained for each subject in every session via the pre-activity MRIs. A zero-minute time point was created for the walk duration data by defining the compressive strain at zero minutes of walking to be zero for all subjects. The 95% confidence interval for the strains about this zero-minute time point was calculated as follows. First, the resting cartilage thickness was calculated (per subject) for each session. Next, these resting thicknesses were averaged across sessions to yield a single representative resting thickness per subject. The standard error of these resting thicknesses was also calculated per subject. Each subject’s standard error was then expressed as a percentage of their mean representative resting thickness. Finally, the mean standard error (expressed as a percent) across all 10 subjects was calculated. This value was used to calculate the 95% confidence interval across all subjects for the zero-minute time point in the same fashion as it was calculated across all subjects for every other duration.

## Supplementary information

Supplementary Information
