# Supplementary material for: A New Stress Test for Knee Joint Cartilage
Source: Sci Rep. 2019 Feb 19;9:2283. doi: 10.1038/s41598-018-38104-2 (PMC6381136; doi:10.1038/s41598-018-38104-2)
Supplement: Supplementary file 1 — Supplementary Information [file 41598_2018_38104_MOESM1_ESM.docx]

**A New Stress Test for Knee Joint Cartilage – Supplementary File**

Chinmay S. Paranjape^*1^, Hattie C. Cutcliffe^*1,2^, Steven C. Grambow^3^, Gangadhar M. Utturkar^1^,
Amber T. Collins^1^, William E. Garrett^1^, Charles E. Spritzer^4^, Louis E. DeFrate^1,2,5^

^1^Department of Orthopaedic Surgery, ^2^Department of Biomedical Engineering,

^3^Department of Biostatistics and Bioinformatics, ^4^Department of Radiology

Duke University, Durham, NC, ^5^Department of Mechanical Engineering and Materials Science,

Duke University, Durham, NC

^*^These authors are co-first authors on this manuscript and contributed equally to its writing.

Corresponding Author:

Louis E. DeFrate, Ph.D.

Frank H. Bassett III, M.D. Associate Professor in Orthopaedic Surgery

Associate Professor in Biomedical Engineering

Duke University Medical Center

Medical Sciences Research Building I

203 Research Drive, Room 375

DUMC Box 3093, Durham, NC 27710

Phone: 919-681-9959

Email: lou.defrate@duke.edu

**Supplementary File**

The following plots illustrate all data points (in addition to the mean and 95% confidence intervals) for Figures 2 and 3 of the main text.


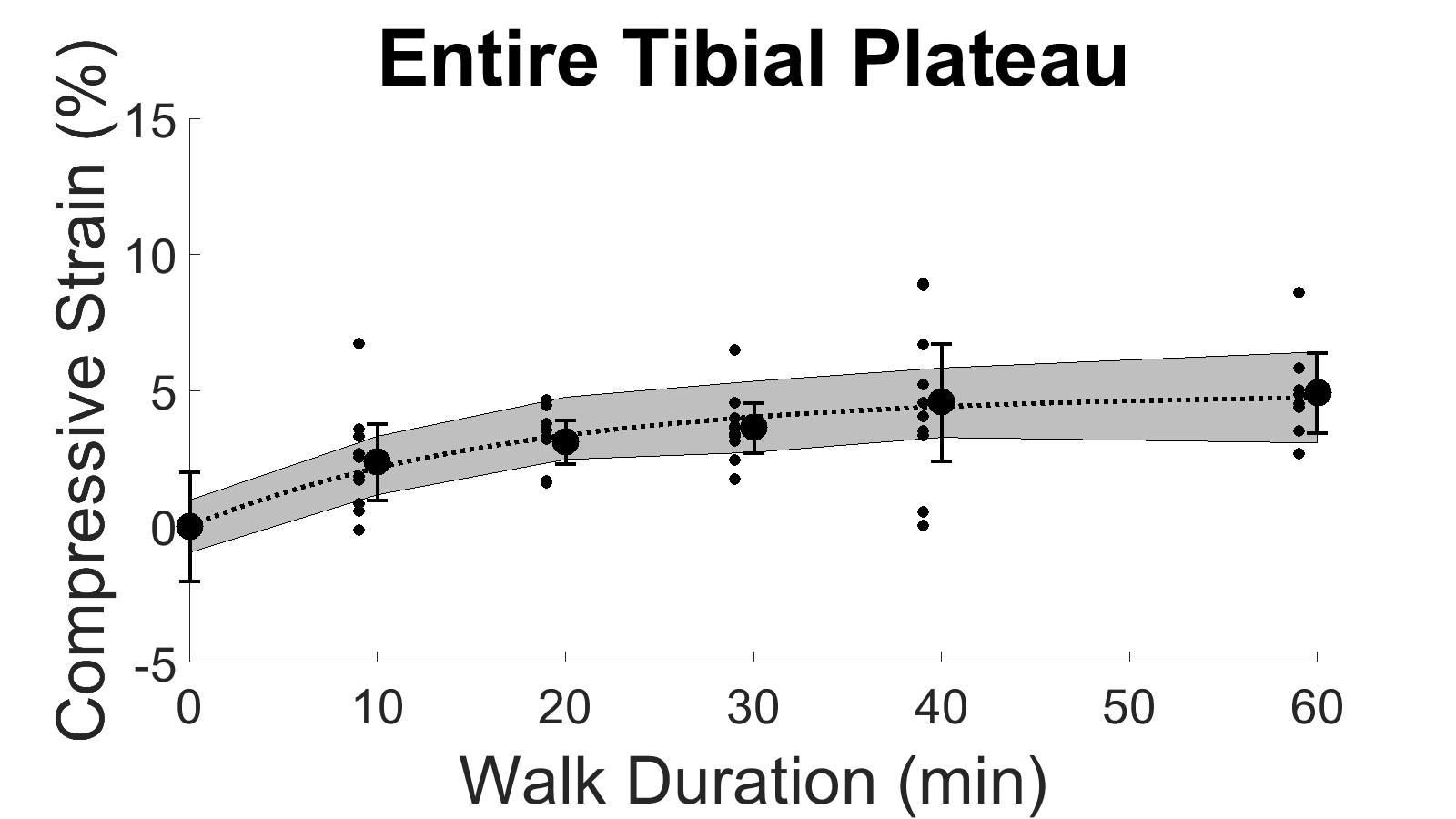


Figure 2a: Overall compressive strain (mean, 95% confidence interval) in tibial cartilage as a function of walk duration, for a fixed normalized walking speed (Fr = 0.25); sample size (n) = 10 for all time points except 60 min where n = 8; compressive strain significantly increased (Spearman correlation, p < 0.05) with increasing walk duration in a nonlinear fashion (lines represent the NLME fits of the Kelvin-Voigt two-parameter creep model to the duration data while shading represents the 95% prediction interval for the median of this fit).


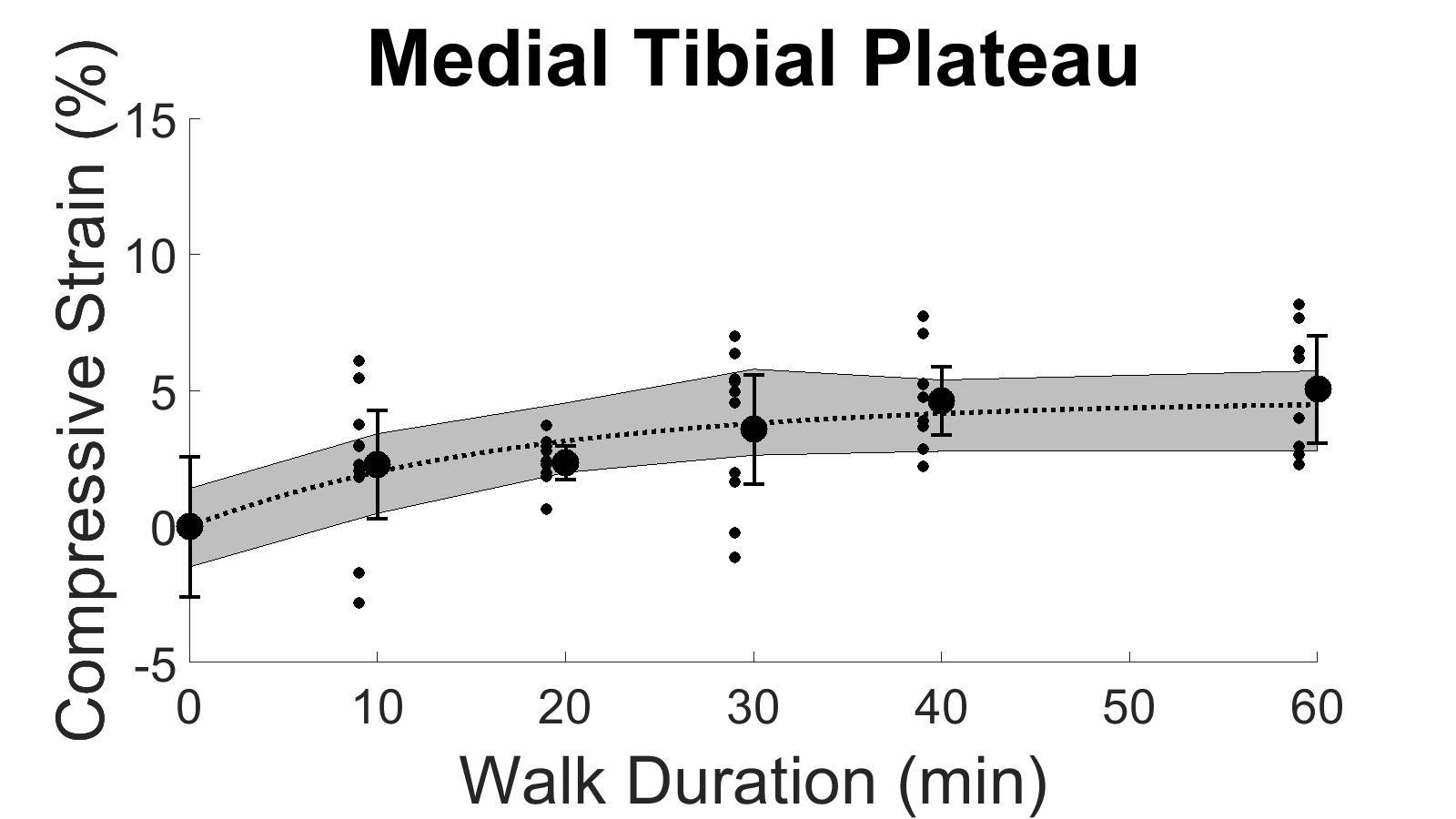


Figure 2b: Medial compartment compressive strain (mean, 95% confidence interval) in tibial cartilage as a function of walk duration, for a fixed normalized walking speed (Fr = 0.25); sample size (n) = 10 for all time points except 60 min where n = 8; compressive strain significantly increased (Spearman correlation, p < 0.05) with increasing walk duration in a nonlinear fashion (lines represent the NLME fits of the Kelvin-Voigt two-parameter creep model to the duration data while shading represents the 95% prediction interval for the median of this fit).


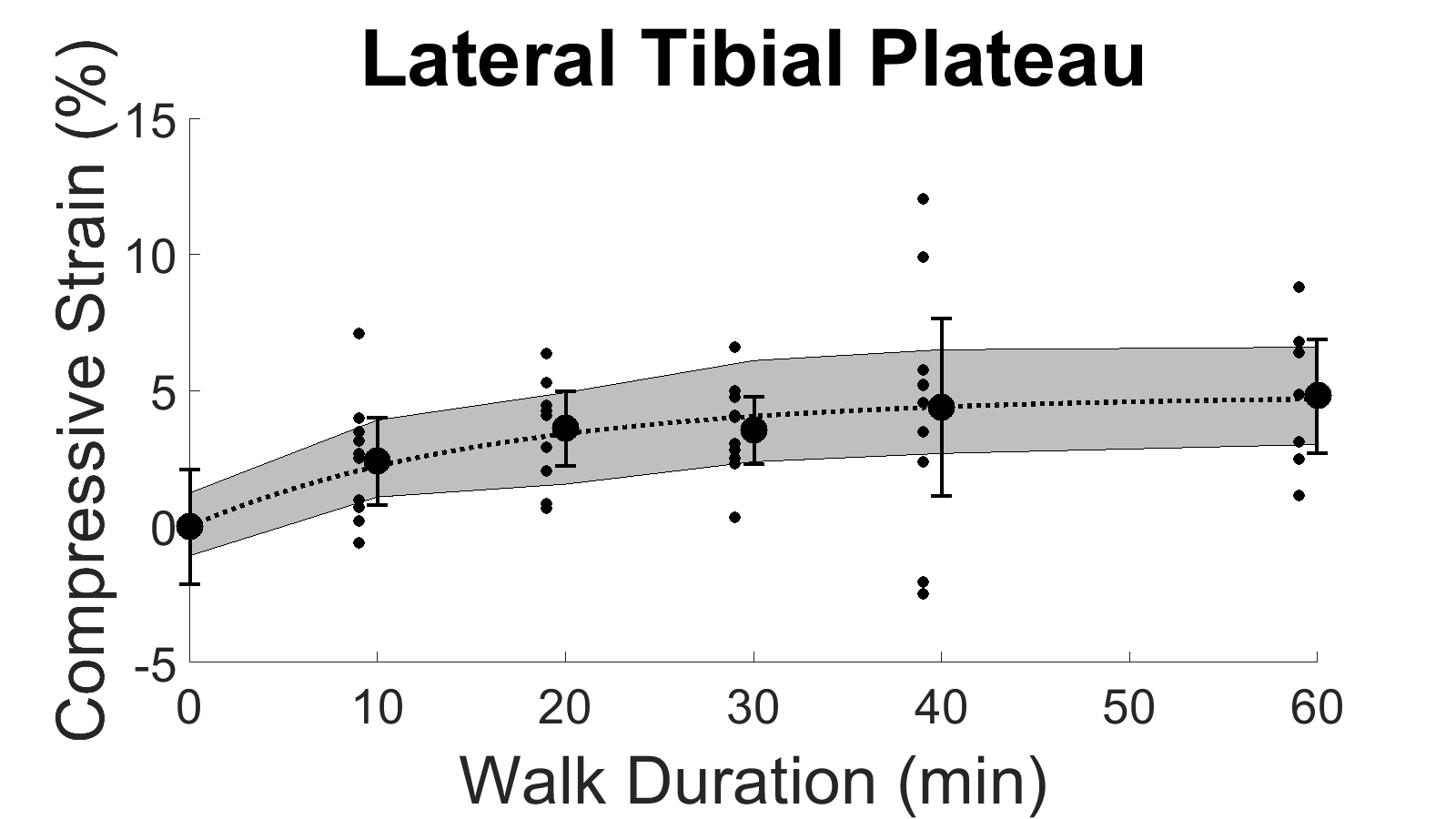


Figure 2c: Lateral compartment compressive strain (mean, 95% confidence interval) in tibial cartilage as a function of walk duration, for a fixed normalized walking speed (Fr = 0.25); sample size (n) = 10 for all time points except 60 min where n = 8; compressive strain significantly increased (Spearman correlation, p < 0.05) with increasing walk duration in a nonlinear fashion (lines represent the NLME fits of the Kelvin-Voigt two-parameter creep model to the duration data while shading represents the 95% prediction interval for the median of this fit).


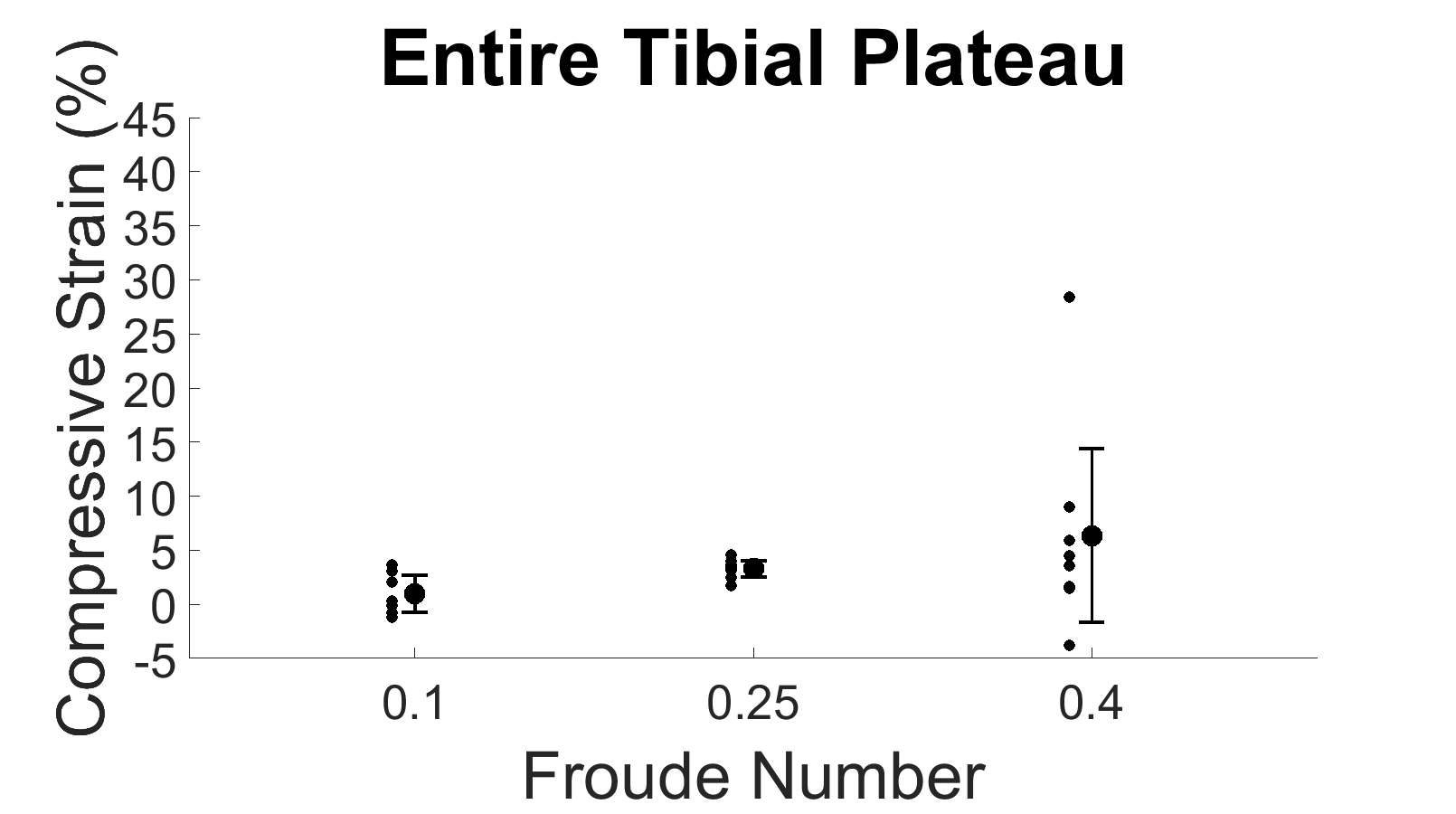


Figure 3a: Overall compressive strain (mean, 95% confidence interval) in tibial cartilage as a function of normalized walking speed (Froude Number, Fr), for a fixed walk duration (30 minutes); sample size (n) = 7 for Fr = 0.1, n = 10 for Fr = 0.25, n = 8 for Fr = 0.40; compressive strain significantly increased (linear mixed model, p < 0.05) with increased normalized walking speed for the overall tibia and medial tibial plateau, but not for the lateral tibial plateau.


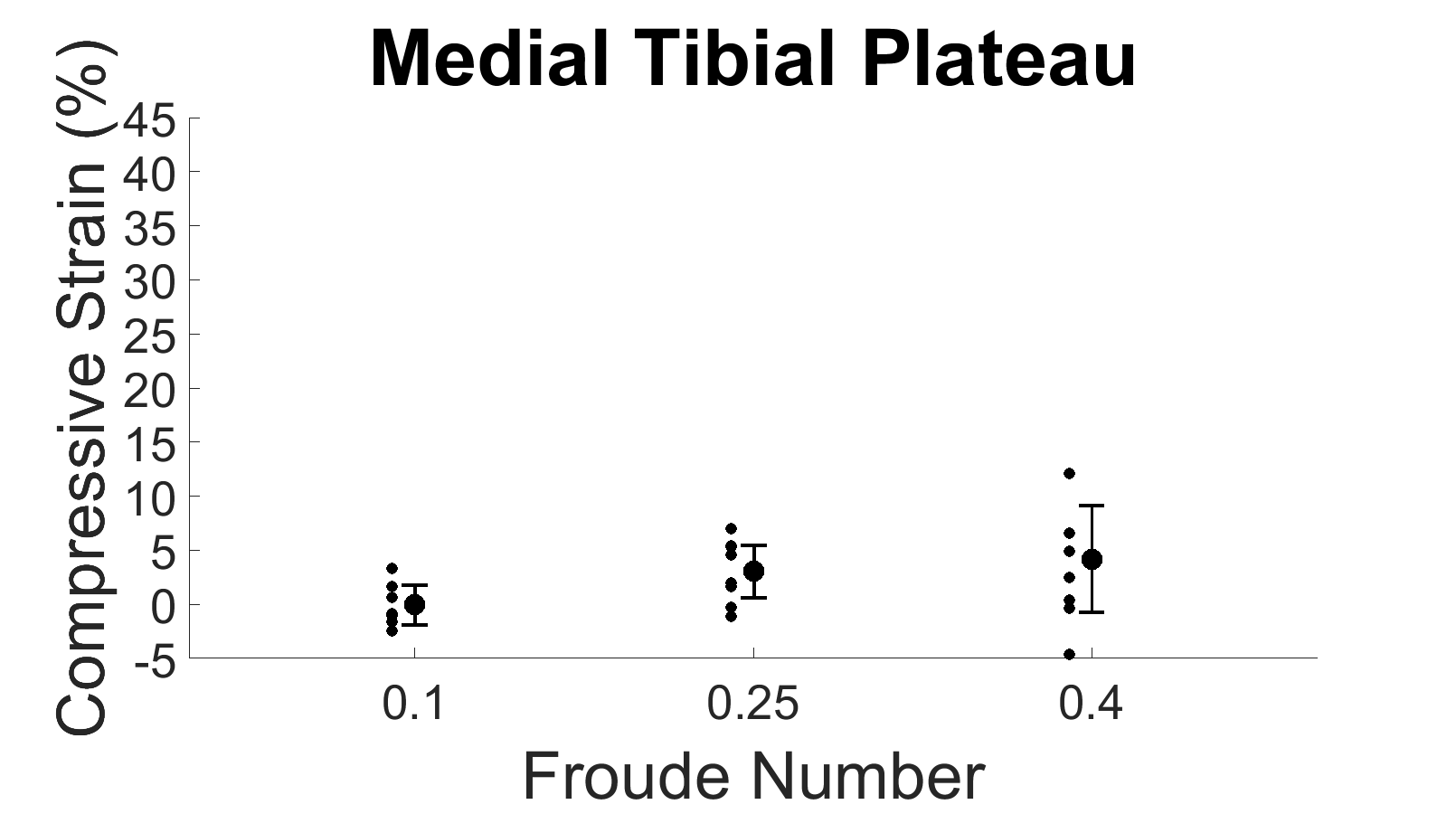


Figure 3b: Medial compartment compressive strain (mean, 95% confidence interval) in tibial cartilage as a function of normalized walking speed (Froude Number, Fr), for a fixed walk duration (30 minutes); sample size (n) = 7 for Fr = 0.1, n = 10 for Fr = 0.25, n = 8 for Fr = 0.40; compressive strain significantly increased (linear mixed model, p < 0.05) with increased normalized walking speed for the overall tibia and medial tibial plateau, but not for the lateral tibial plateau.


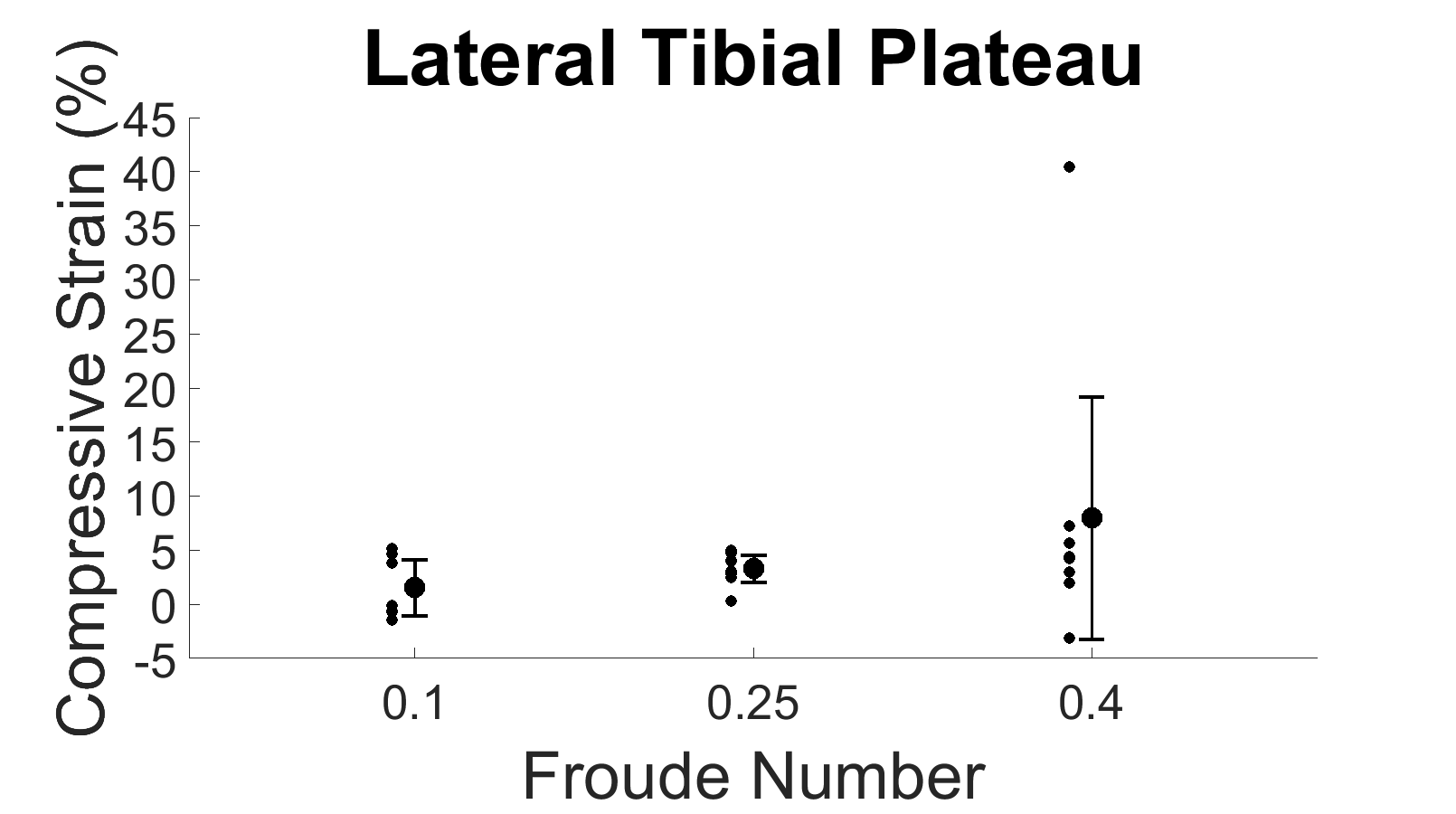


Figure 3c: Lateral compartment compressive strain (mean, 95% confidence interval) in tibial cartilage as a function of normalized walking speed (Froude Number, Fr), for a fixed walk duration (30 minutes); sample size (n) = 7 for Fr = 0.1, n = 10 for Fr = 0.25, n = 8 for Fr = 0.40; compressive strain significantly increased (linear mixed model, p < 0.05) with increased normalized walking speed for the overall tibia and medial tibial plateau, but not for the lateral tibial plateau.

The following plots illustrate the NLME model evaluation tools provided by the saemix package (Comets et al. 2017) for the Kelvin-Voigt two-parameter creep model fit of the duration data. Model evaluations are given for the fits to the overall strain, medial strain, and lateral strain. The Q-Q plots and frequency vs NPDE (normalized prediction distribution errors) plots indicate that the model fits the data well, while the NPDE vs X and NPDE vs Predicted Y plots indicate that the error is randomly distributed, further indicating good model diagnostics.

Reference:

Comets, E., Lavenu, A. & Lavielle, M. Parameter estimation in nonlinear mixed effect models using saemix, and R implementation of the SAEM algorithm. *Journal of Statistical Software* **80**, 1-41, doi:10.18637/jss.v080.i03 (2017).

Overall strain:

Medial Strain:


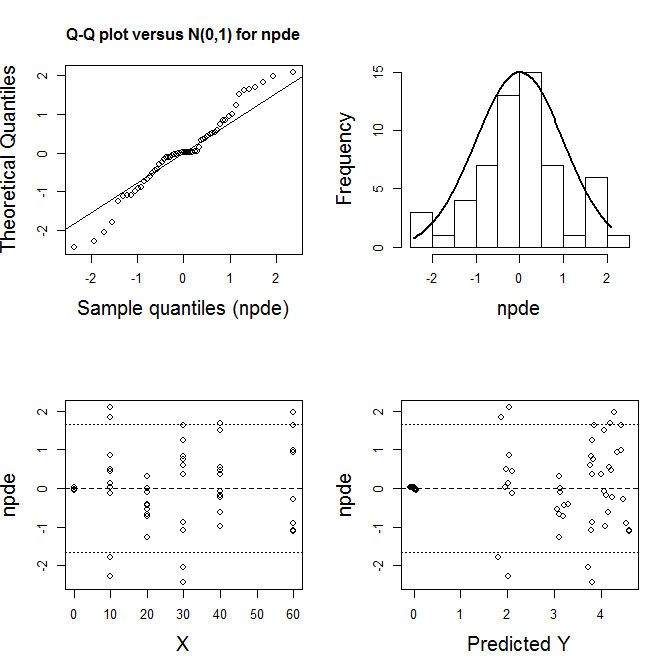


Lateral Strain:

**
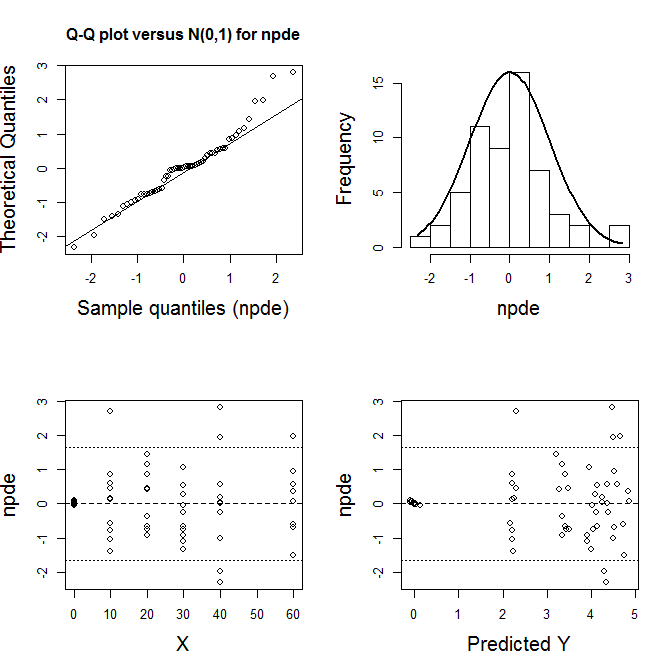
**
